# Expression of HIF‑α and their association with clinicopathological parameters in clinical renal cell carcinoma

**DOI:** 10.48101/ujms.v129.9407

**Published:** 2024-03-21

**Authors:** Raviprakash T. Sitaram, Börje Ljungberg

**Affiliations:** 1 Department of Odontology, Umeå University, Umeå, Sweden; 2 Department of Surgical and Perioperative Sciences, Urology and Andrology, Umeå University, Umeå, Sweden

**Keywords:** renal cell carcinoma, ccRCC, non-ccRCC, HIF-1α, HIF-2α, HIF-3α, prognosis, and tumor stage

## Abstract

**Objectives:**

This study aimed to assess the cellular localization and expression levels of hypoxia-inducible factor (HIF) -α proteins (specifically HIF-1α, HIF-2α, and HIF-3α) that play a role in the hypoxia pathway and to determine their correlation with clinicopathological parameters and patient survival in renal cell carcinoma (RCC).

**Materials and methods:**

Tissue microarray (TMA) with cores from 150 clear cell RCCs and 31 non-ccRCC samples. HIF-1α, HIF-2α, and HIF-3α antibodies were used for immunohistochemistry (IHC) of TMA to evaluate the cellular localization and expression levels of HIF-α proteins, specifically in relation to the hypoxia pathway.

**Results:**

The expression levels of the HIF-α proteins were higher in the nucleus than in the cytoplasm. Furthermore, the nuclear expression levels of all HIF-α proteins were significantly higher in clear cell RCC (ccRCC) than in non-ccRCC. Cytoplasmic HIF-3α expression was also higher in ccRCC than in non-ccRCC, whereas cytoplasmic HIF-1α and HIF-2α expression levels were similar between the different RCC types. In ccRCC, nuclear HIF-1α expression levels correlated with both nuclear HIF-2α and HIF-3α levels, whereas cytoplasmic HIF-3α expression levels were associated with HIF-1α only.

In non-ccRCC, there was a positive correlation observed between nuclear HIF-1α and HIF-3α expression, but no correlation was found with HIF-2α. In patients with ccRCC, the nuclear expressions of HIF-1α and HIF-3α was significantly associated with cancer-specific survival (CSS) in univariate analysis. This association was no longer evident in multivariate analysis. Notably, there was no correlation observed between nuclear HIF-2α expression and CSS in these patients. In contrast, cytoplasmic expression levels showed no association with CSS.

**Conclusion:**

The expression levels of the three primary HIF-α proteins were found to be higher in the nucleus than in the cytoplasm. Furthermore, the results indicated that HIF-3α and HIF-1α expression levels were significant univariate factors associated with CSS in patients with clear cell RCC. These results highlight the critical role that HIF-3α and HIF-1α play in the hypoxia pathway.

## Introduction

Renal cell carcinoma (RCC) is a type of cancer that accounts for approximately 3% of all adult cancers and is more commonly found in Western countries (1). It affects males more often than females, with the peak incidence occurring in people aged 60–70 (2). RCC is classified into different types based on its histopathological and genetic characteristics, with the most common types being clear cell RCC (ccRCC), papillary RCC (pRCC), and chromophobe RCC (chRCC) (3). Common genetic abnormalities in ccRCC include loss of heterozygosity (LOH), hypermethylation, mutations, and deletions in the 3p chromosomal region (4). These aberrations in chromosome 3p cause inactivation of the von Hippel-Lindau (VHL) gene, leading to decreased transcription of VHL protein (pVHL) (5). In contrast, non-ccRCC (pRCC and chRCC) rarely shows chromosome 3p aberrations (6). The hallmark of pRCC is germline mutations in the MET proto-oncogene, which activates MET signalling to promote tumor and cell motility (7). chRCC is associated with Brit-Hogg-Dube syndrome, and the most common genetic alterations include the LOH of chromosomes 1, 2, 6, 10, 13, 17, and 21 (7).

Due to the involvement of different genes and signalling pathways, ccRCC and non-ccRCC behave differently with respect to tumor progression and spread (8). Under normal conditions, the VHL protein (pVHL) functions as an adaptor protein like the E3-ubiquitin ligase complex, and aids in the degradation of hypoxia-inducible factor-α (HIF)-α subunits by ubiquitination (9). Tumors activate the hypoxia response pathway through HIF-α when there is a lack of intracellular oxygen to maintain oxygen availability (10). HIF-α comprises three unstable subunits: HIF-1α, HIF-2α, and HIF-3α, which are encoded by HIF1A, EPAS1, and HIF3A, respectively. Although HIF-1α and HIF-2α have similar protein structures and amino acid sequences, they have different functions (11, 12). HIF-1α and HIF-2α are associated with tumorigenesis, metastasis, and disease progression in RCC (12–15). The effects of HIF-3α are still not fully understood and there is less amino acid sequence similarity between HIF-1α and HIF-2α (16). It undergoes alternative splicing to generate variants (17, 18). The HIF-3α4 splice variant exerts a dominant-negative effect on hypoxic responses (17, 19). HIF-3α is a positive transcriptional regulator of several downstream molecules. However, the role of HIF-3α in ontogeny remains unclear (20).

This study aimed to elucidate the protein expression levels and cellular localization of HIF-3α, HIF-1α, and HIF-2α and their association with clinicopathological parameters in RCC.

## Materials and methods

### Patient and public involvement

All patients provided informed consent and since January 2000 a signed informed consent to participate in the study was used. Patients were informed that the studies included survival information, laboratory values, measurements of tumor variables, and genetic changes. The Institutional Review Board and Ethics Committee of Northern Sweden approved the study. Patients were informed that they could leave the study for any reason at any time.

### Tissue samples

Multiple tumor and kidney cortex tissue samples were obtained from surgically removed tumor-bearing kidneys, formalin-fixed, and histologically examined. A total of 181 patients were surgically treated with radical or partial nephrectomy between 1988 and 2009 at the University Hospital in Umeå, Sweden (21). The RCC type was defined according to the Heidelberg classification, tumor stage according to the TNM classification (22), and nuclear grade according to the Fuhrman classification (23). The distribution of patient characteristics in relation to the RCC type is summarized in Table 1. TNM stage groups I and II were collected and stages III and IV were collected for statistical analysis. Similarly, Grades 1 and 2 and grades 3 and 4 were gathered. The patients were followed up with a scheduled follow-up program.

**Table 1:** Distribution of patients’ characteristics is shown in relation to RCC type in 181 patients with RCC.

Variable	ccRCC	Non-ccRCC	RCC
	*n* = 150	(*n* = 31)	(*n* = 181)
Age (years)			
Mean	65.8	64.00	65.58
Median (range)	67 (34–87)	65 (25–82)	67 (25–87)
Gender			
Men	87 (58.0%)	17 (54.8%)	104 (57.4%)
Women	63 (42.0%)	14 (45.2%)	77 (42.5%)
T-stage			
T1	48 (32.0%)	10 (32.3%)	58 (32.0%)
T2	26 (17.3%)	7 (22.6%)	33 (18.2%)
T3	31 (20.7%)	10 (32.3%)	41 (22.7%)
T4	45 (30.0%)	4 (12.9%)	49 (27.5%)
N-stage			
No	105 (70%)	27 (87.1%)	132 (72.92%)
N1	45 (30%)	4 (12.9%)	49 (27.07%)
Survival			
Alive	52 (33.2%)	9 (29%)	61 (32.2%)

### Tissue microarray construction

Four representative tumors and two kidney cortex cores measuring 0.6 mm in diameter were placed in a newly prepared recipient paraffin block from formalin-fixed and paraffin-embedded tissue blocks. The tissue microarray (TMA) blocks were sliced into 4 µm sections and treated according to standard procedures, including deparaffinization and rehydration. A representative slice of each TMA block was stained with hematoxylin and eosin. The stained TMA sections were reviewed and confirmed by a clinical pathologist. Four representative tumors and two kidney cortex cores measuring 0.6 mm in diameter were placed in a newly prepared recipient paraffin block from formalin-fixed and paraffin-embedded tissue blocks. The TMA blocks were sliced into 4 µm sections and treated according to standard procedures, including deparaffinization and rehydration. A representative slice of each TMA block was stained with hematoxylin and eosin. Stained TMA sections were reviewed and confirmed by a clinical pathologist.

### Immunohistochemical staining

The TMA sections were treated with citrate buffer (pH 6) for antigen retrieval, followed by endogenous peroxidase blocking with methanol (200 mL) containing 3 mL of 40% H202 for 20 min. Sections were incubated with primary antibodies at the following dilutions: HIF-1α (NB100-132; Novus Biologicals, Cambridge, UK; 1:200), HIF-2α (NB100-134; Novus Biologicals, 1:150), and HIF-3α (ab10134; Abcam, Cambridge, UK; 1:200). EnVision+ Dual-link Single Reagent (HRP. Rabbit/Mouse; Agilent CA, USA) was used as the secondary antibody. Finally, the sections were visualized using diaminobenzidine/ H202 and counter-stained with hematoxylin. Immunohistochemistry (IHC) was performed on 150 ccRCC and 31 non-ccRCC samples. Owing to the loss of cores during IHC, 149 ccRCCs were analyzed for HIF-1α, 149 for HIF-2α, and 148 for HIF-3α. TMA sections were treated with citrate buffer (pH 6) for antigen retrieval, followed by endogenous peroxidase blocking with methanol (200 mL) containing 3 mL of 40% H202 for 20 min. The sections were incubated with primary antibodies at the following dilutions: HIF-1α (NB100-132; Novus Biologicals, Cambridge, UK; 1:200), HIF-2α (NB100-134; Novus Biologicals, 1:150), and HIF-3α (ab10134; Abcam, Cambridge, UK; 1:200). EnVision+ Dual-link Single Reagent (HRP. Rabbit/Mouse; Agilent CA, USA) was used as the secondary antibody. Finally, the sections were visualized using diaminobenzidine/H202 and counterstained with hematoxylin. IHC was performed on 150 ccRCC samples and 31 non-ccRCC samples. Owing to the loss of cores during IHC, 149 ccRCCs were analyzed for HIF-1α, 149 for HIF-2α, and 148 for HIF-3α.

### Scoring of protein of expression in cytoplasm and nucleolus

A Panoramic 250 scanner (3DHistech, Budapest, Hungary) was used to digitally scan the IHC-stained TMA slides at a magnification of 40 ×. Furthermore, we employed QuPath version 0.2.0-m429,30, an open-source image analysis platform (Center for Cancer Research & Cell Biology, University of Edinburgh), to arrange disordered IHC-stained TMAs. All cores were evaluated during the scoring process to manually exclude invalid cores (<10% of the tumor per core or artifacts). A simple, automated, and semi-assisted method using QuPath was used for TMA quantification. After several steps and subsequent validations, the desired threshold for the positive cells was selected for each marker. Staining vectors were automatically analyzed for each scanned TMA slide, followed by total tissue area detection, separation of tumor from non-tumor areas in each core, and automatic cellular detection. Positive cells were assigned using the optical density threshold of the selected cells, tested on each core, and applied to the entire array after validation by an expert pathologist.

The histochemical score (H-score) measures the intensity of staining. The H-score was obtained by calculating the sum of the percentage of staining multiplied by the corresponding intensity, and was used as the expression level.

### Statistical analysis

SPSS Statistics 27.0 (IBM) was used for the statistical analysis. The Mann–Whitney U test was used to compare the variable levels between the two independent groups. In addition, Cox regression analysis was used for multivariable analysis. Kaplan–Meier curves illustrating survival times were analyzed using the log-rank test. For all tests, a two-sided *P*-value less than 0.05 was considered significant.

## Results

### Localization of HIF-1a, HIF-2a, and HIF-3a

**Figure 1:**
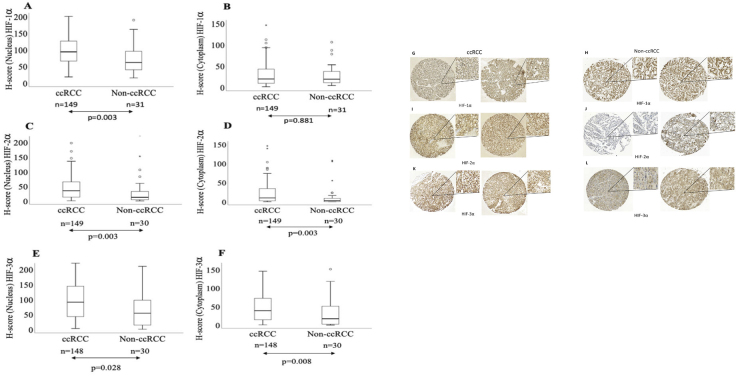
Box plots representation of expression levels of (A) nuclear HIF-1α, (B) cytoplasmic HIF-1α, (C) nuclear HIF-2α, (D) cytoplasmic HIF-2α (E) nuclear HIF-3α, and (F) cytoplasmic HIF-3α, in ccRCC patients compared with non-ccRCC; Representative stained tissues cores of ccRCC and non-ccRCC after IHC assay with (G and H) HIF-1α, (I and J) HIF-2α, (K and L) HIF-3α in ccRCC and non-ccRCC, respectively.

**Figure 2:**
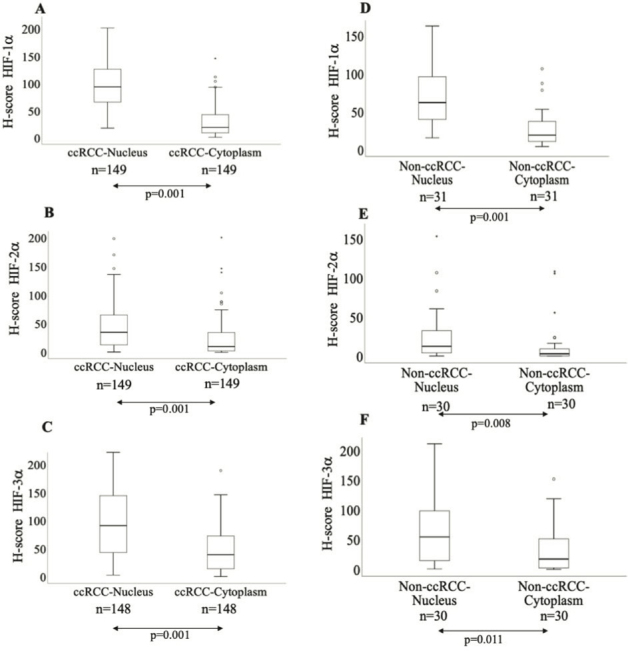
Box plots showing the comparison of expression levels of (A) nuclear HIF-1α and cytoplasmic HIF-1α, (B) nuclear HIF-2α and cytoplasmic HIF-2α, (C) nuclear HIF-3α and cytoplasmic HIF-3α in ccRCC patients; Box plots showing the comparison of expression levels of (D) nuclear HIF-1α and cytoplasmic HIF-1α, (E) nuclear HIF-2α and cytoplasmic HIF-2α, (F) nuclear HIF-3α and cytoplasmic HIF-3α in non-ccRCC patients.

The expression levels of nuclear HIF-1α, HIF-2α, and HIF-3α proteins were significantly higher in ccRCC than in non-ccRCC tissues (Figures 1A, C, and E). Interestingly, both ccRCC and non-ccRCC showed similar cytoplasmic HIF-1α levels. However, the expression levels of cytoplasmic HIF-2α and HIF-3α were significantly higher in ccRCC than in non-ccRCC tissues (Figures 1B, D, and F). Representative tissue sections of ccRCC and non-ccRCC stained with HIF-1α, HIF-2α, and HIF-3α are shown in Figure 1G–L. Moreover, in ccRCC, the expression levels of HIF-1α, HIF-2α, and HIF-3α are significantly higher in the nucleus than in the cytoplasm. Similarly, in non-ccRCC tissues, all three HIF-α proteins showed significantly higher expression levels in the nucleus than in the cytoplasm (Figure 2A–F).

### Association between nuclear and cytoplasmic HIF-1a, HIF-2a, and HIF-3a protein levels, and clinicopathological parameters

The expression levels of nuclear HIF-1α, HIF-2α, and HIF-3α in ccRCC did not vary based on age, gender (data not shown) or between tumor grade in ccRCC (Table 2, Supplementary Figure 1A–F). However, there was a significant difference in nuclear HIF-2α expression between advanced T stages (II–IV, *n* = 102) and lower T stages (I–II, *n* = 47, *P* = 0.033). In patients with ccRCC, it was found that the expression of HIF-1α was significantly lower in TNM stage I compared with stage IV in the nucleus and the expression of HIF-2α was significantly lower in TNM stage I compared with stage II in both the nucleus and cytoplasm. However, no significant difference in expression was observed between stage I and stages III and IV (as shown in Supplementary Figure 2A, C and D).

**Table 2:** HIF-1α, HIF-2α, and HIF-3α H-expression levels in relation to tumor grade, TNM stage, and tumor size in 150 patients with ccRCC. Expression values are presented as mean, median, and IQR.

Variable	HIF-1α			*P*		HIF-2α		*P*		HIF-3α		*P*
	*n*	Median (IQR)	Mean		*n*	Median (IQR)	Mean		*n*	Median (IQR)	Mean	
Tumor grade 1 – 2 3 – 4	58 91	87.3 (63.4) 95.9 (57.9)	75.7 74.4	0.837	58 91	30.8 (45.5) 38.4 (53.7)	72.6 76.5	0.591	59 89	76.1 (113.1) 93.3 (88.4)	73.7 75.0	0.852
TNM -stage I – II	73	85.3 (68.9)	69.7	0.145	73	34.4 (50.7)	74.2	0.817	73	87.4 (92.6)	71.7	0.440
III – IV	76	101.0 (52.4)	80.1		76	37.3 (54.6)	75.8		75	99.3 (113.7)	77.2	
Tumor size ≤ 70 ≥ 70	66 83	84.4 (65.9) 99.3 (54.0)	68.6 88.1	0.106	66 83	28.3 (43.7) 43.4 (56.9)	66.6 81.7	0.035*	66 82	80.2 (115.5) 94.6 (88.4)	73.0 75.7	0.701

Note: ccRCC, clear cell renal cell carcinoma; n, number of patients; Tumor grade, Fuhrman grade classification; TNM stage, TNM stage groups; IQR, the interquartile range. Significant *P*-values are given in

* **bold**. There was one (0.7%) missing tumor for HIF-3α analysis.

Additionally, there was a significant correlation between nuclear HIF-2α expression and tumor size (*P* = 0.035), whereas not with HIF-1α (*P* = 0.106) and HIF-3α (*P* = 0.701) (Table 2). There was no association between cytoplasmic HIF-1α, HIF-2α, or HIF-3α expression and the clinicopathological parameters (data not shown).

In non-ccRCC, neither nuclear nor cytoplasmic HIF-1α, HIF-2α, or HIF-3α exhibited differences in their expression levels with any clinicopathological parameter (data not shown).

### Correlations between levels of HIF-α proteins

In ccRCC, nuclear HIF-1α expression levels correlated significantly with both nuclear HIF-2α and HIF-3α levels, whereas HIF-1α correlated with HIF-2α only (Table 3). Cytoplasmic HIF-1α expression levels significantly correlated with cytoplasmic HIF-2α and HIF-3α expression levels (Table 4).

**Table 3:** Correlations between nuclear HIF-1α, HIF-2α, and HIF-3α expression levels in relation to RCC type (ccRCC and non-ccRCC, respectively).

Variables	ccRCC		Non-ccRCC	
	HIF-2α (Nucleus)	HIF-3α (Nucleus)	HIF-2α (Nucleus)	HIF-3α (Nucleus)
**HIF-1**α **(Nucleus)**	*P* = 0.002*, *r* = 0.253	*P* = 0.001*, *r* = 0.268	*P* = 0.423, *r* = -0.152	*P* < 0.001*, *r* = 0.689
	*n* = 149	*n* = 148	*n* = 30	*n* = 30
**HIF-2**α **(Nucleus)**		*P* = 0.755, *r* = 0.026		*P* = 0.956, *r* = 0.010
		*n* = 148		*n* = 30

* Spearman’s correlation analyses (Significant at *P* < 0.05)

**Table 4:** Correlations between cytoplasm HIF-1α, HIF-2α, and HIF-3α expression levels in relation to RCC type (ccRCC and non-ccRCC, respectively).

Variables	ccRCC		Non-ccRCC	
	HIF-2α (Cytoplasm)	HIF-3α (Cytoplasm)	HIF-2α (Cytoplasm)	HIF-3α (Cytoplasm)
**HIF-1α (Cytoplasm)**	*P* < 0.001*, *r* = 0.43	*P* = 0.005*, *r* = 0.231	*P* = 0.040, *r* = 0.340	*P* = 0.736, *r* = 0.064
	*n* = 149	*n* = 148	*n* = 30	*n* = 30
				
**HIF-2α (Cytoplasm)**		*P* = 0.031*, *r* = 0.178		*P* = 0.385, *r* = 0.165
		*n* = 148		*n* = 30

* Spearman’s correlation analysis (Significant at *P* < 0.05)

In contrast, in non-ccRCC, nuclear HIF-1α correlated with nuclear HIF-3α expression levels, and cytoplasmic HIF-1α correlated with HIF-2α expression levels only (Table 4).

### Relation between HIF-1α, HIF-2α, and HIF-3α localization and cancer-specific survival

**Figure 3:**
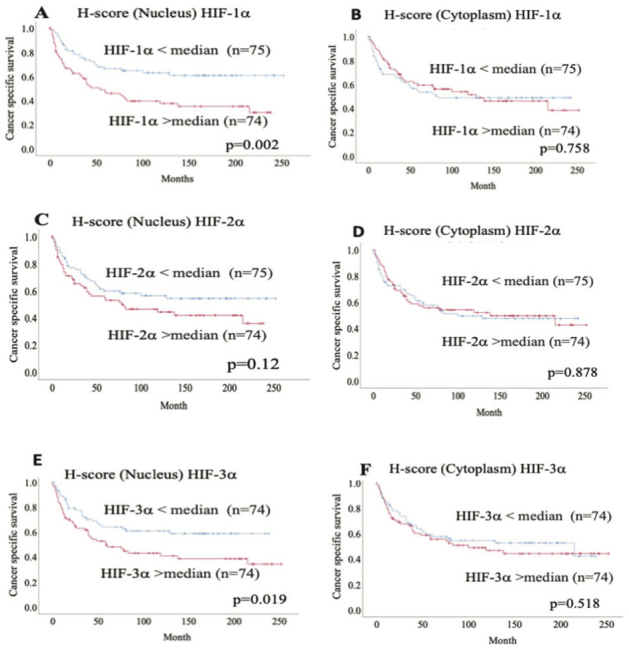
Kaplan–Meier plots showing cancer-specific survival curves of ccRCC (A) nuclear HIF-1α, (B) cytoplasmic HIF-1α (cytoplasm), (C) nuclear HIF-2α, (D) cytoplasmic HIF-2α, (E) nuclear HIF-3α and (F) cytoplasmic HIF-3α.

In ccRCC, patients with higher nuclear HIF-1α (*P* = 0.002) and HIF-3α (*P* = 0.019) (> median value) expression levels had significantly shorter survival than those with lower levels (< median value) and cancer-specific survival (CSS), whereas nuclear HIF-2α expression (*P* = 0.12) had no association with CSS. In Cox regression analysis, both HIF-1α and HIF-3α protein expression were significantly associated with CSS in univariate analysis but did not remain significant after adjusted analysis (Table 5). Cytoplasmic HIF-α protein expression was not associated with CSS (Figure 3A–F). In non-ccRCC patients, HIF-α protein expression levels, neither in the nucleus nor in the cytoplasm, were associated with CSS (data not shown).

**Table 5:** Results from Cox regression analysis of factors important for cancer-specific survival in 149 patients with clear cell renal cell carcinoma, adjusted for age, gender, tumor size (mm), tumor grade, tumor stage, and HIF-1α, HIF-2α, and HIF-3α nuclear and cytoplasmic expression levels, respectively.

Predictor	Unadjusted			Adjusted^a^		
	RR	95% CI	*P*	RR	95% CI	*P*
Age continous	0.998	0.985–1.012	0.821	0.984	0.958–1.011	0.242
Female vs. Male	0.832	0.611–1.134	0.244	1.721	0.954–3.106	0.071
Tumor Size (mm)	1.013	1.010–1.017	**< 0.001**	1.006	0.998–1.014	0.114
Grade (1, 2 vs. 3,4)	2.352	1.928–2.868	**< 0.001**	1.511	1.108–2.059	**0.009**
Stage (I, II vs. III,IV)	8.365	5.659–12.366	**< 0.001**	8.543	4.183–17.447	**< 0.001**
HIF-1α-Nuclear	2.106	1.306–3.397	**0.002**	1.919	0.864–4.261	0.109
HIF-1α-Cytoplasm	1.428	0.897–2.275	0.133	1.010	0.805–1.267	0.930
HIF-2α-Nuclear	0.988	0.619–1.576	0.959	0.956	0.485–1.887	0.898
HIF-2α-Cytoplasm	1.039	0.633–1.705	0.881	0.891	0.423–1.875	0.760
HIF-3α-Nuclear	1.872	1.165–3.009	**0.010**	1.242	0.628–2.458	0.533
HIF-3α-Cytoplasm	1.635	1.028–2.600	**0.038**	1.504	0.740–3.058	0.260

**Note:** CI, confidence interval; RR, risk ratio; Tumor Size, tumor size in mm; Grade, tumor grade; Stage, TNM tumor stage; Significant *P*-values are given in **bold**.

## Discussion

This study found a significant association between CSS and the nuclear expression levels of HIF-1α and HIF-3α, suggesting that these proteins are significantly involved in angiogenesis and proliferation in ccRCC. The hypoxia response pathway is activated by hypoxia or VHL mutations in the tumor microenvironment (24, 25). Various experimental models have shown a critical role for HIF-1α and HIF-2α in tumor progression and patient survival (26, 27). Limited data are available on HIF-3α, and its function as a regulator of the hypoxia response pathway remains unclear.

In this study, we report that HIF-α proteins are predominantly localized in the nucleus and have higher expression levels in ccRCC than in non-ccRCC tissues. These findings are in line with those of previous studies (26, 28). One reason for the high nuclear localization of HIF-α could be that hypoxia triggers the expression of HIF-α and its downstream targets, very-low-density receptor (VLDL-R) and HIG 2. As a result, lipid content accumulates in the cytoplasm and HIF-α proteins accumulate in the nuclear compartment (29, 30). Accumulation in the nucleus is further supported by the fact that HIF-α proteins possess a basic helix–loop–helix domain that heterodimerises with the stable aryl hydrocarbon receptor nuclear translocator HIF-1β (31). We found no association between nuclear HIF-α protein expression levels and age, gender, and tumor grade, while nuclear HIF-2α expression levels were associated with advanced T stage and tumor size. This is consistent with previous studies showing higher HIF-2α expression at later stages (32, 33). Furthermore, we found that higher nuclear expression levels of HIF-1α were associated with poorer CSS in ccRCC patients. This is consistent with a previous meta-analysis that showed that high nuclear HIF-1α expression in ccRCC is associated with unfavorable prognosis (34). The interaction of HIF-1α with various signalling pathways is responsible for its effect on patient survival, as reported in previous studies (35, 36).

Similar to earlier reports, we observed no association between nuclear or cytoplasmic HIF-2α expression and survival in patients with ccRCC (26, 37, 38). In contrast to our results, a previous study reported that high cytoplasmic HIF-2α expression levels were associated with poor survival (38). However, the reason for this discrepancy remains unclear. This difference might be due to the use of different methods to analyze HIF-2α expression levels; other cancers, such as breast carcinoma, were included in that study. Likewise, high HIF-2α expression levels in tumor-associated macrophages (TAMA) indicate a poor prognosis in patients with breast cancer patients (39, 40). Furthermore, in non-ccRCC patients, we found no association between HIF-α protein expression levels and any clinicopathological parameter or patient survival. A previous study hypothesized that only the ccRCC phenotype in humans had a cause-effect association with HIF-α (28). The lack of relevance of hypoxia to non-ccRCC ontogenesis could be a plausible explanation.

Our study demonstrated the feasibility of analyzing HIF-3α protein expression levels in tissue samples obtained from a patient with RCC. We observed a significant association between nuclear HIF-3α expression and CSS in patients with ccRCC. In addition, the levels of HIF-3α expression were considerably higher in the nucleus than in the cytoplasm and higher in ccRCC than in non-ccRCC. These findings are noteworthy because earlier data on the relationship between HIF-3α and clinicopathological variables in RCC are sparse. Despite sharing a similar set of target genes, HIF-3α did not compete with the other two HIF-α counterparts in an experimental environment (41). Various splice variants of HIF-3α exhibit diverse functions: a short HIF-3α splice variant acts as a dominant-negative inhibitor of the hypoxia response and a long HIF-3α performs transactivation activity (17, 42).

Similar to a previous study, we found a significant correlation between nuclear HIFα-proteins and ccRCC (26). This correlation is attributable to the crucial function of HIF-α proteins in the hypoxia response pathway, which regulates genes involved in cellular proliferation and survival, thereby promoting ccRCC growth and progression.

This study concluded that HIF-α protein expression levels were significantly higher in ccRCC than in non-ccRCC, with higher expression levels in the nucleus than in the cytoplasm. In addition, HIF-1α and HIF-3α nuclear expression levels were significantly associated with CSS in patients with ccRCC in univariate analysis but not HIF-2 α, implying that the major HIF-α proteins have different biological features that are crucial for tumor progression. However, in multivariate analysis, neither HIF-1α nor HIF-3α nuclear expression levels remained independent prognostic factors.

## Ethics approval statement

All samples were obtained after obtaining informed consent from patients. The Institutional Review Board approved the study and the ethics committee of Northern Sweden.

## Disclosure statement

The authors declare no competing interest.

## Supplementary Material



## Data sharing statement

The datasets relevant to this study are submitted with article and supplementary data.

## References

[1] Ferlay J, Colombet M, Soerjomataram I, Dyba T, Randi G, Bettio M, et al. Cancer incidence and mortality patterns in Europe: estimates for 40 countries and 25 major cancers in 2018. Eur J Cancer. 2018;103:356–87. doi: 10.1016/j.ejca.2018.07.005 30100160

[2] Ljungberg B, Albiges L, Abu-Ghanem Y, Bedke J, Capitanio U, Dabestani S, et al. European Association of Urology guidelines on renal cell carcinoma: The 2022 update. Eur Urol. 2022;82(4):389–410. 10.1016/j.eururo.2022.03.006 35346519

[3] Ricketts CJ, De Cubas AA, Fan H, Smith CC, Lang M, Reznik E, et al. The cancer genome atlas comprehensive molecular characterization of renal cell carcinoma. Cell Rep. 2018;23:313–26 e315. doi: 10.1016/j.celrep.2018.03.075 29617669 PMC6075733

[4] Kovacs G. Molecular genetics of human renal cell tumours. Nephrol Dial Transplant. 1996;11(Suppl 6):62–5. 10.1093/ndt/11.supp6.62 9044331

[5] Batavia AA, Schraml P, Moch H. Clear cell renal cell carcinoma with wild-type von Hippel-Lindau gene: a non-existent or new tumour entity? Histopathology. 2019;74:60–7. doi: 10.1111/his.13749 30565303

[6] Sanjmyatav J, Hauke S, Gajda M, Hartmann A, Moch H, Meyer B, et al. Establishment of a multicolour fluorescence in situ hybridisation-based assay for subtyping of renal cell tumours. Eur Urol. 2013;64:689–91. 10.1016/j.eururo.2013.06.007 23790440

[7] Yap NY, Rajandram R, Ng KL, Pailoor J, Fadzli A, Gobe GC. Genetic and chromosomal aberrations and their clinical significance in renal neoplasms. Biomed Res Int. 2015;2015:476508. doi: 10.1155/2015/476508 26448938 PMC4584050

[8] Cheville JC, Lohse CM, Zincke H, Weaver AL, Blute ML. Comparisons of outcome and prognostic features among histologic subtypes of renal cell carcinoma. Am J Surg Pathol. 2003;27:612–24. 10.1097/00000478-200305000-00005 12717246

[9] Frew IJ, Krek W. pVHL: a multipurpose adaptor protein. Sci Signal. 2008;1:e30. doi: 10.1126/scisignal.124pe30 18560019

[10] Rankin EB, Giaccia AJ, Schipani E. A central role for hypoxic signaling in cartilage, bone, and hematopoiesis. Curr Osteoporos Rep. 2011;9:46–52. doi: 10.1007/s11914-011-0047-2 21360287 PMC4012534

[11] Hu CJ, Wang LY, Chodosh LA, Keith B, Simon MC. Differential roles of hypoxia-inducible factor 1alpha (HIF-1alpha) and HIF-2alpha in hypoxic gene regulation. Mol Cell Biol. 2003;23:9361–74. doi: 10.1128/mcb.23.24.9361-9374.2003 14645546 PMC309606

[12] Klatte T, Seligson DB, Riggs SB, Leppert JT, Berkman MK, Kleid MD, et al. Hypoxia-inducible factor 1 alpha in clear cell renal cell carcinoma. Clin Cancer Res. 2007;13:7388–93. doi: 10.1158/1078-0432.CCR-07-0411 18094421

[13] Biswas S, Charlesworth PJ, Turner GD, Leek R, Thamboo PT, Campo L, et al. CD31 angiogenesis and combined expression of HIF-1alpha and HIF-2alpha are prognostic in primary clear-cell renal cell carcinoma (CC-RCC), but HIFalpha transcriptional products are not: implications for antiangiogenic trials and HIFalpha biomarker studies in primary CC-RCC. Carcinogenesis. 2012;33:1717–25. doi: 10.1093/carcin/bgs222 22777959

[14] Gordan JD, Simon MC. Hypoxia-inducible factors: central regulators of the tumor phenotype. Curr Opin Genet Dev. 2007;17:71–7. doi: 10.1016/j.gde.2006.12.006 17208433 PMC3215290

[15] Minardi D, Lucarini G, Santoni M, Mazzucchelli R, Burattini L, Conti A, et al. Survival in patients with clear cell renal cell carcinoma is predicted by HIF-1alpha expression. Anticancer Res. 2015;35:433–8. 25550584

[16] Gu YZ, Moran SM, Hogenesch JB, Wartman L, Bradfield CA. Molecular characterization and chromosomal localization of a third alpha-class hypoxia inducible factor subunit, HIF3alpha. Gene Exp. 1998;7:205–13. PMC6151950 9840812

[17] Pasanen A, Heikkila M, Rautavuoma K, Hirsila M, Kivirikko KI, Myllyharju J. Hypoxia-inducible factor (HIF)-3alpha is subject to extensive alternative splicing in human tissues and cancer cells and is regulated by HIF-1 but not HIF-2. Int J Biochem Cell Biol. 2010;42:1189–200. doi: 10.1016/j.biocel.2010.04.008 20416395

[18] Prabhakar NR, Semenza GL. Adaptive and maladaptive cardiorespiratory responses to continuous and intermittent hypoxia mediated by hypoxia-inducible factors 1 and 2. Physiol Rev. 2012;92:967–1003. doi: 10.1152/physrev.00030.2011 22811423 PMC3893888

[19] Heikkila M, Pasanen A, Kivirikko KI, Myllyharju J. Roles of the human hypoxia-inducible factor (HIF)-3alpha variants in the hypoxia response. Cell Mol Life Sci. 2011;68:3885–901. doi: 10.1007/s00018-011-0679-5 21479871 PMC11114783

[20] Li QF, Wang XR, Yang YW, Lin H. Hypoxia upregulates hypoxia inducible factor (HIF)-3alpha expression in lung epithelial cells: characterization and comparison with HIF-1alpha. Cell Res. 2006;16:548–58. doi: 10.1038/sj.cr.7310072 16775626

[21] Svenson U, Ljungberg B, Roos G. Telomere length in peripheral blood predicts survival in clear cell renal cell carcinoma. Cancer Res. 2009;69:2896–901. doi: 10.1158/0008-5472.CAN-08-3513 19318563

[22] Kovacs G, Akhtar M, Beckwith BJ, Bugert P, Cooper CS, Delahunt B, et al. The Heidelberg classification of renal cell tumours. J Pathol. 1997;183:131–3. doi: 10.1002/(SICI)1096-9896(199710)183:2<131::AID-PATH931>3.0.CO;2-G 9390023

[23] Fuhrman SA, Lasky LC, Limas C. Prognostic significance of morphologic parameters in renal cell carcinoma. Am J Surg Pathol. 1982;6:655–63. doi: 10.1097/00000478-198210000-00007 7180965

[24] Haase VH. Hypoxia-inducible factors in the kidney. Am J Physiol Renal Physiol. 2006;291:F271–81. doi: 10.1152/ajprenal.00071.2006 16554418 PMC4232221

[25] Semenza GL. Targeting HIF-1 for cancer therapy. Nat Rev Cancer. 2003;3:721–32. doi: 10.1038/nrc1187 13130303

[26] Kroeze SG, Vermaat JS, Van Brussel A, Van Melick HH, Voest EE, Jonges TG, et al. Expression of nuclear FIH independently predicts overall survival of clear cell renal cell carcinoma patients. Eur J Cancer. 2010;46:3375–82. doi: 10.1016/j.ejca.2010.07.018 20709525

[27] Schodel J, Grampp S, Maher ER, Moch H, Ratcliffe PJ, Russo P, et al. Hypoxia, hypoxia-inducible transcription factors, and renal cancer. Eur Urol. 2016;69:646–57. doi: 10.1016/j.eururo.2015.08.007 26298207 PMC5012644

[28] Toth K, Chintala S, Rustum YM. Constitutive expression of HIF-alpha plays a major role in generation of clear-cell phenotype in human primary and metastatic renal carcinoma. Appl Immunohistochem Mol Morphol. 2014;22:642–7. doi: 10.1097/PAI.0000000000000012 25046225 PMC4184929

[29] Gimm T, Wiese M, Teschemacher B, Deggerich A, Schodel J, Knaup KX, et al. Hypoxia-inducible protein 2 is a novel lipid droplet protein and a specific target gene of hypoxia-inducible factor-1. FASEB J. 2010;24:4443–58. doi: 10.1096/fj.10-159806 20624928

[30] Sundelin JP, Stahlman M, Lundqvist A, Levin M, Parini P, Johansson ME, et al. Increased expression of the very low-density lipoprotein receptor mediates lipid accumulation in clear-cell renal cell carcinoma. PLoS One. 2012;7:e48694. doi: 10.1371/journal.pone.0048694 23185271 PMC3501495

[31] Dengler VL, Galbraith M, Espinosa JM. Transcriptional regulation by hypoxia inducible factors. Crit Rev Biochem Mol Biol. 2014;49:1–15. doi: 10.3109/10409238.2013.838205 24099156 PMC4342852

[32] Raval RR, Lau KW, Tran MG, Sowter HM, Mandriota SJ, Li JL, et al. Contrasting properties of hypoxia-inducible factor 1 (HIF-1) and HIF-2 in von Hippel-Lindau-associated renal cell carcinoma. Mol Cell Biol. 2005;25:5675–86. doi: 10.1128/MCB.25.13.5675-5686.2005 15964822 PMC1157001

[33] Mandriota SJ, Turner KJ, Davies DR, Murray PG, Morgan NV, Sowter HM, et al. HIF activation identifies early lesions in VHL kidneys: evidence for site-specific tumor suppressor function in the nephron. Cancer Cell. 2002;1:459–68. doi: 10.1016/s1535-6108(02)00071-5 12124175

[34] Fan Y, Li H, Ma X, Gao Y, Chen L, Li X, et al. Prognostic significance of hypoxia-inducible factor expression in renal cell carcinoma: a PRISMA-compliant systematic review and meta-analysis. Medicine (Baltimore). 2015;94:e1646. doi: 10.1097/MD.0000000000001646 26402839 PMC4635779

[35] Tumkur Sitaram R, Landstrom M, Roos G, Ljungberg B. Significance of PI3K signalling pathway in clear cell renal cell carcinoma in relation to VHL and HIF status. J Clin Pathol. 2020;74(4):216–22. doi: 10.1136/jclinpath-2020-206693 32467322

[36] Tumkur Sitaram R, Landström, M., Roos, G., Ljungberg, B. Role of Wnt signaling pathways in clear cell renal cell carcinoma pathogenesis in relation to VHL and HIF status. Clin Oncol Res. 2020;3. doi: 10.31487/j.COR.2020.03.09 32467322

[37] Sandlund J, Ljungberg B, Wikstrom P, Grankvist K, Lindh G, Rasmuson T. Hypoxia-inducible factor-2alpha mRNA expression in human renal cell carcinoma. Acta Oncol. 2009;48:909–14. doi: 10.1080/02841860902824891 19322701

[38] Moreno Roig E, Yaromina A, Houben R, Groot AJ, Dubois L, Vooijs M. Prognostic role of hypoxia-inducible factor-2alpha tumor cell expression in cancer patients: a meta-analysis. Front Oncol. 2018;8:224. doi: 10.3389/fonc.2018.00224 29942795 PMC6004384

[39] Leek RD, Lewis CE, Whitehouse R, Greenall M, Clarke J, Harris AL. Association of macrophage infiltration with angiogenesis and prognosis in invasive breast carcinoma. Cancer Res. 1996;56:4625–29. 8840975

[40] Leek RD, Talks KL, Pezzella F, Turley H, Campo L, Brown NS, et al. Relation of hypoxia-inducible factor-2 alpha (HIF-2 alpha) expression in tumor-infiltrative macrophages to tumor angiogenesis and the oxidative thymidine phosphorylase pathway in Human breast cancer. Cancer Res. 2002;62:1326–9. 11888900

[41] Tolonen JP, Heikkila M, Malinen M, Lee HM, Palvimo JJ, Wei GH, et al. A long hypoxia-inducible factor 3 isoform 2 is a transcription activator that regulates erythropoietin. Cell Mol Life Sci. 2020;77:3627–642. doi: 10.1007/s00018-019-03387-9 31768607 PMC7452874

[42] Zhang P, Bai Y, Lu L, Li Y, Duan C. An oxygen-insensitive Hif-3alpha isoform inhibits Wnt signaling by destabilizing the nuclear beta-catenin complex. Elife. 2016;5:e08996. doi: 10.7554/eLife.08996 26765566 PMC4769163

