# Supplementary material for: Expression of HIF‑α and their association with clinicopathological parameters in clinical renal cell carcinoma
Source: Ups J Med Sci. 2024 Mar 21;129:10.48101/ujms.v129.9407. doi: 10.48101/ujms.v129.9407 (PMC10989218; doi:10.48101/ujms.v129.9407)

Supplementary Figure -1

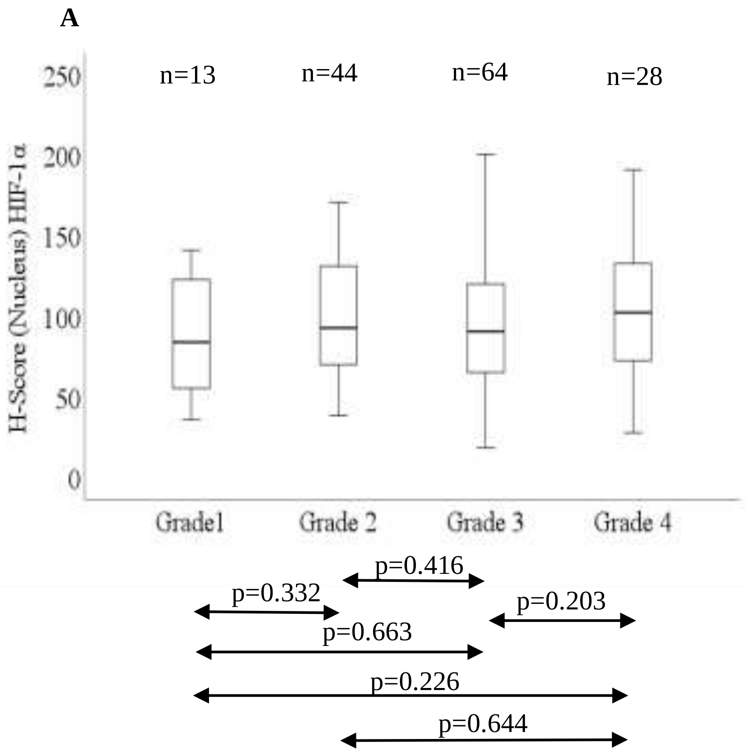

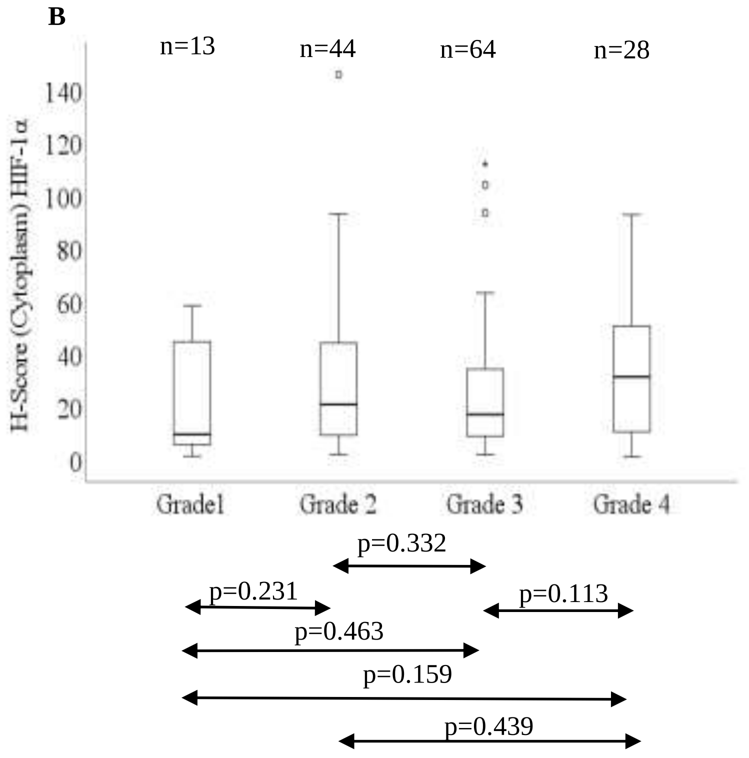

Supplementary Figure -1

C

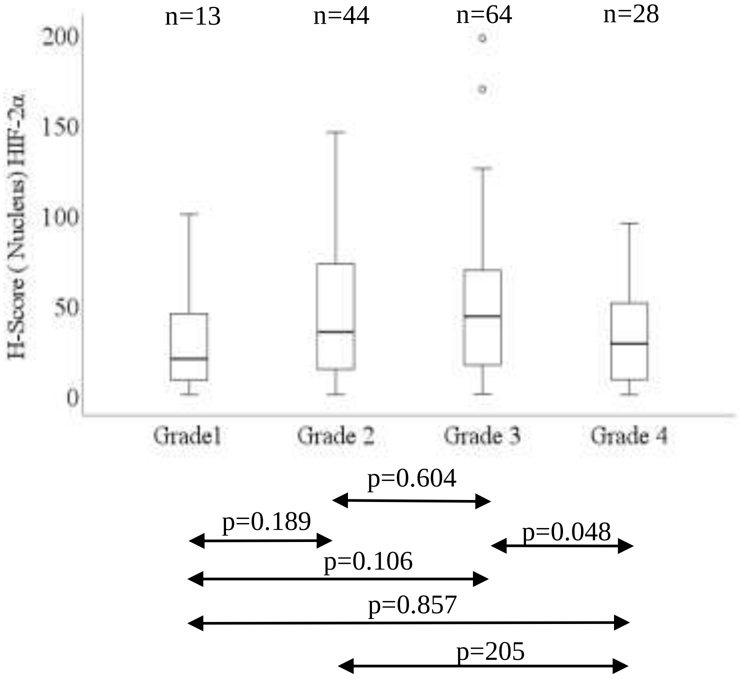

D

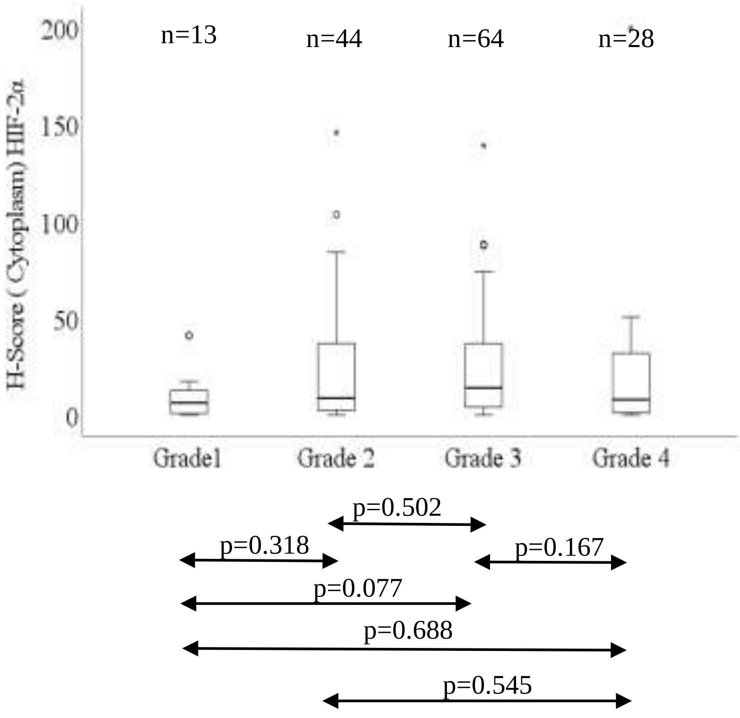

Supplementary Figure 1

E

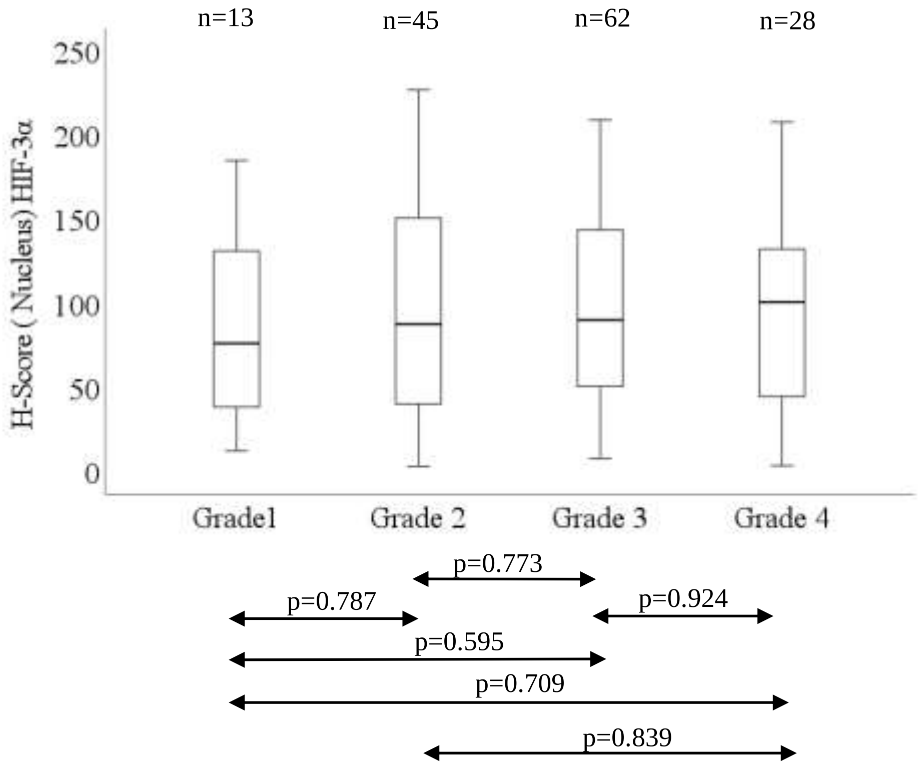

F

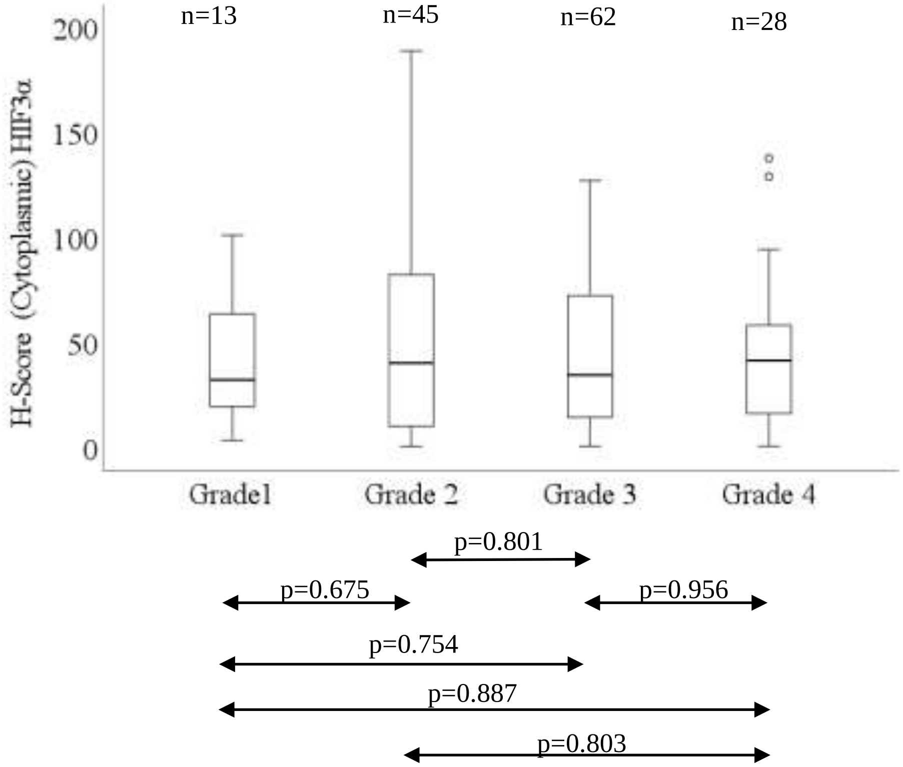

Supplementary Figure -2

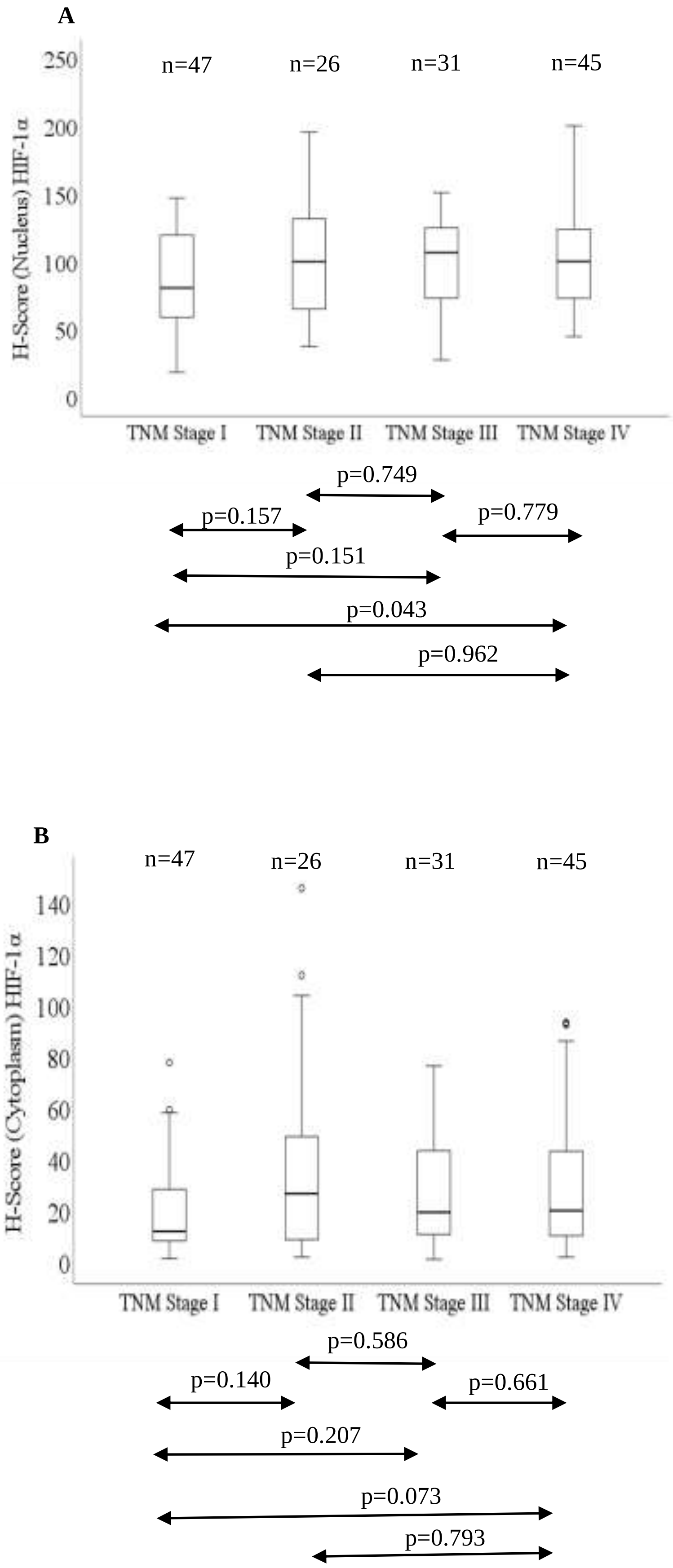

Supplementary Figure -2

C

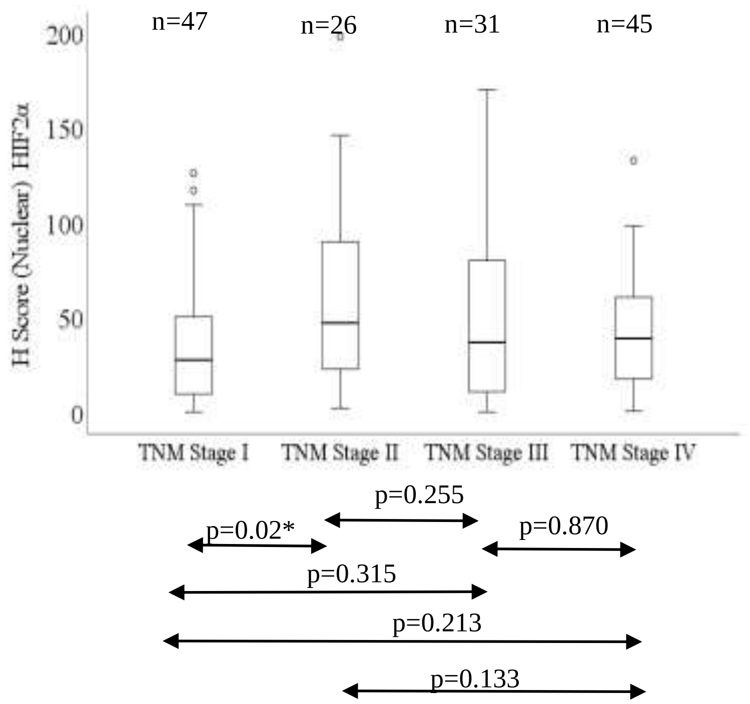

D

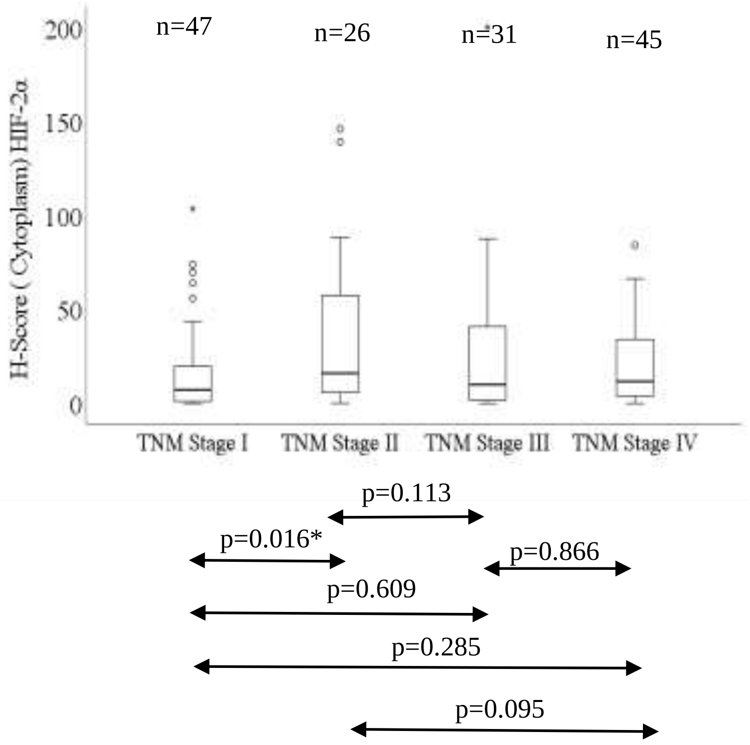

Supplementary Figure -2

E

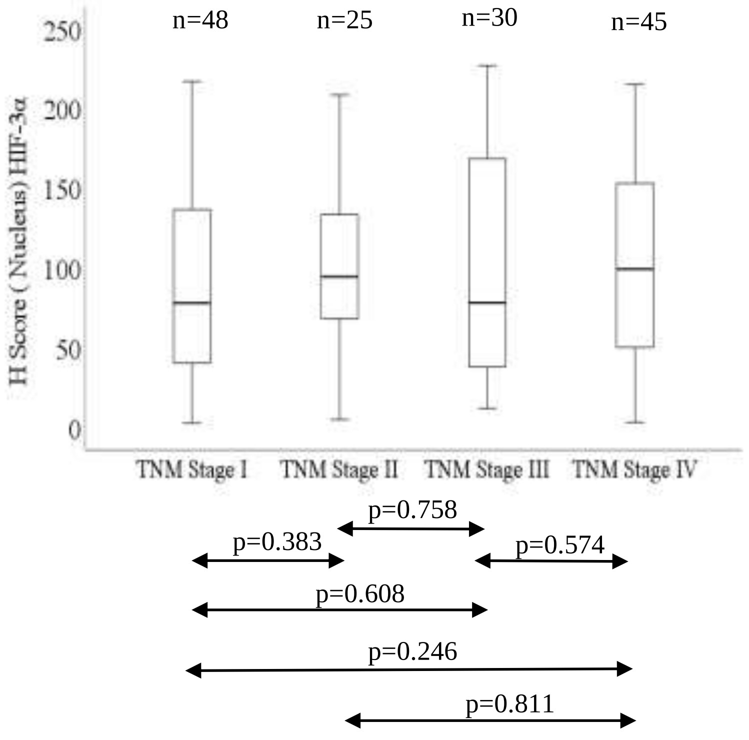

F

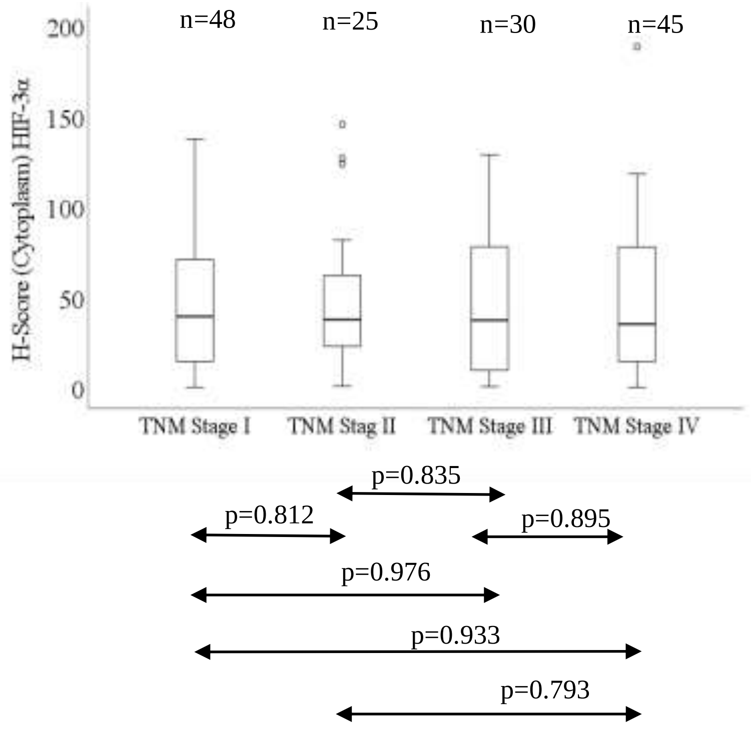

Supplement: Supplementary file 1 [file UJMS-129-9407-s1.pdf]
